# Application of hair HPG axis hormone levels before onset in predicting the risk of stroke

**DOI:** 10.3389/fneur.2025.1595429

**Published:** 2025-08-28

**Authors:** Jiao Yang, Shuhan Li, Xuan Li, Tiantian Wang, Huihua Deng, Yuanyuan Jia, Dianhuai Meng

**Affiliations:** ^1^Rehabilitation Center, The First Affiliated Hospital with Nanjing Medical University, Nanjing, China; ^2^School of Biological Science and Medical Engineering, Southeast University, Nanjing, China; ^3^Department of Rehabilitation Medicine, The Eighth Affiliated Hospital of Southern Medical University (The First People’s Hospital of Shunde Foshan), Foshan, China

**Keywords:** stroke, hypothalamic–pituitary-gonadal axis, hormones, testosterone, progesterone

## Abstract

**Background:**

There have been many studies on the relationship between sex hormones and stress, mood, blood pressure, etc., but its impact on the incidence of stroke remains unknown.

**Objective:**

To investigate the expression levels of hypothalamic–pituitary-gonadal (HPG) axis related hormones such as testosterone (T) and progesterone (P) in hair before stroke and their effects on the risk of stroke.

**Methods:**

48 patients with stroke were recruited from November 2022 to May 2023 as the observation group and 35 healthy subjects were recruited as the control group. There was no obvious difference in age, gender and BMI between the two groups (*p* > 0.05). T and P levels in hair were tested by LC-MS/MS, and the correlation with the risk of stroke was analyzed.

**Results:**

The T and P levels of hair before the onset of stroke in the observation group were significantly lower than those in the control group (*p* < 0.01). The T level of men’s hair before the onset of stroke in the observation group was significantly lower than those in the control group (*p* < 0.05). The T and P levels of women’s hair before the onset of stroke in the observation group were significantly lower than those in the control group (*p* < 0.01). The results of ROC curve showed that the cut-off value of T level in men’s hair before the onset of stroke was 4.35 pg/mg, the AUC was 0.690 (0.545, 0.835), the sensitivity was 62.50%, and the specificity was 82.61%. The cut-off value of T level in women’s hair before the onset of stroke was 5.00 pg/mg, the AUC was 0.818 (0.658, 0.978), the sensitivity was 75.00%, and the specificity was 83.33%. The cut-off value of P level in women’s hair before the onset of stroke was 8.00 pg/mg, the AUC was 0.891 (0.754, 1.000), the sensitivity was 81.25%, and the specificity was 100.00%.

**Conclusion:**

This preliminary report is the first to suggest that HPG axis hormones such as T and P in hair could have predictive value in screening for stroke risk.

## 1 Introduction

Cerebrovascular diseases have now ascended to the foremost cause of mortality in China, with stroke constituting the single most prevalent etiology of disability, exerting a substantial burden on both individuals and society (1). Stroke is a preventable and manageable disease, and early screening combined with active intervention can markedly enhance patient prognosis (2).

Emerging evidence suggests associations between hypothalamic–pituitary-gonadal (HPG) axis hormones including testosterone (T), estradiol (E_2_), and progesterone (P) and stroke incidence (3, 4). Haya et al. (5) conducted a 29-year follow-up study involving 4,615 adult males and 4,724 adult females to investigate endogenous hormone profiles, revealing that extremely low serum T concentrations were significantly associated with elevated ischemic stroke risk in males. However, this study faced methodological limitations, including the absence of precise temporal data on stroke onset in community-based populations, which has led to the need for studies in larger samples. Furthermore, the requirement for continuous serological monitoring and extended follow-up until stroke occurrence resulted in prolonged study duration, substantial costs, and potential confounding from unadjusted variables such as aging.

Hormone levels in human hair serve as endogenous biomarkers for retrospective assessment of hypothalamic–pituitary-adrenocortical (HPA) and HPG axis activity (6). The measurement of hormone levels in hair is retrospective, with 1 cm of hair corresponding to approximately 1 month of hormonal accumulation, and a reliable retrospective window extending up to 6 months (7). Compared to traditional biological matrices such as blood, serum, and urine, hair hormone analysis offers distinct advantages, including cumulative representation of long-term exposure, non-invasive sampling, and the ability to reflect baseline levels over extended periods (similar to how hemoglobin A1c is used to assess serum glucose levels) (7–9). While clinical studies on hair hormones have proliferated (10, 11), current research predominantly focuses on associations between hair hormone levels and psychiatric, emotional, and sleep-related disorders. For instance, Wright et al. (12) demonstrated in a systematic review that cortisol quantification in hair can evaluate chronic stress exposure in elderly populations. Deng et al. (11) investigated functional characteristics of the HPA and HPG axes, as well as their interplay, in patients with schizophrenia by analyzing expression levels of eight biomarkers in hair samples. Similarly, Wang et al. (13) identified a significant correlation between pre-onset HPA axis hyperactivity and post-stroke emotional disorders.

Current research lacks substantial evidence on the correlation between pre-stroke HPG axis hormone levels in hair and stroke risk. Considering the influence of age and sex on HPG axis activity (14), this study investigates the relationship between the expression levels of HPG axis hormones in the hair and the risk of stroke onset among individuals aged over 50, across different genders. The findings aim to provide novel insights and methodologies for early stroke screening and intervention strategies.

## 2 Materials and methods

This study was reviewed and approved by the Ethics Committee of Jiangsu Provincial People’s Hospital (2022-SR-553), and was prospectively registered with the Chinese Clinical Trial Registry (ChiCTR2200065803). All participants provided written informed consent to participate in the study.

### 2.1 Participants

Study participants included 48 stroke patients (32 males, 16 females) hospitalized at Jiangsu Provincial People’s Hospital and its Qixia Rehabilitation Branch between November 2022 and May 2023. Subjects in the stroke group had a mean age of 64.67 ± 7.53 years, with a mean disease duration of 31.50 days (IQR 33.00). Stroke group included 35 ischemic and 13 hemorrhagic cases. A control group of 35 healthy individuals (23 males, 12 females; mean age 65.69 ± 8.90 years) was recruited. No statistically significant differences were observed between groups in age, sex, or body mass index (BMI) (*p* > 0.05) (Table 1).

**Table 1:** Comparison of general data between the two groups [M (IQR)] or M ± SD.

Basic information	Stroke group (*n* = 48)	Control group (*n* = 35)	*χ*2/t	*p* value
Gender (*n*%)				
Male	32 (66.67)	23 (65.71)	0.008	0.928
Female	16 (33.33)	12 (34.29)		
Age (years)	64.67 ± 7.53	65.69 ± 8.90	−0.563	0.575
BMI (kg/m^2^)	23.59 ± 3.20	24.54 ± 1.99	−1.556	0.124
Duration (days)	31.5 (33.00)	-	-	-
Stroke type				
Ischemic case	35 (72.92)	-	-	-
Hemorrhagic case	13 (27.08)	-	-	-

Inclusion Criteria for Stroke Group were as follows: ① Age 50–80 years; ➁ Stroke diagnosis confirmed by neuroimaging and compliant with diagnostic guidelines established by the Chinese Stroke Society; ➂ First-ever unilateral stroke; ➃ Disease duration ≤ 3 months; ➄ Clinically stable with vital signs within normal ranges; ➅ Postmenopausal status (confirmed by amenorrhea ≥ 12 months) for female participants.

Inclusion Criteria for Control Group were as follows: ① Age 50–80 years; ➁ No history of stroke or major neurological disorders; ➂ Postmenopausal status for female participants.

Exclusion Criteria (Both Groups) were as follows: ① Auditory comprehension deficits or inability to follow verbal commands; ➁ Concurrent with other central nervous system lesions (e.g., traumatic brain injury, brain neoplasms); ➂ History of reproductive system disorders or surgeries; ➃ Substance abuse, hormone therapy within the past year, or chronic alcoholism; ➄ Occipital hair length < 3 cm; ➅ Recent hair treatments affecting analysis.

### 2.2 Sample collection

Hair samples were collected from all subjects on their enrollment day. Hair samples were collected by cutting 1 cm of hair from the occipital region, located at the back of the subject’s head near the scalp. Specimens were wrapped in aluminum foil, labeled, and stored at room temperature protected from light.

### 2.3 Hormonal analysis

Hormonal concentrations in hair were analyzed via high-performance liquid chromatography–tandem mass spectrometry (LC-MS/MS), where an Agilent 1,200 liquid chromatograph (Agilent Technologies, Inc., United States) was combined with an API 3200 Q-TRAP mass spectrometer (Applied Biosystems, Inc., United States), following a previously developed method (15). Hair samples corresponding to the period before the onset of lesions were selected based on the disease course (If the duration of the patient’s disease is 30 days at the time of admission, the 1 cm of hair closest to the scalp is the total amount of hormones accumulated in the hair in the month when the stroke occurred, and the 2 cm close to the scalp is the total amount of hormones accumulated in the hair in the month before the stroke occurred). Hair samples were rinsed with 5 mL of methanol for 2 min, and then heated to 50°C for drying. In a 2 mL centrifuge tube, hair samples were weighed after measuring 1–2 mm in length and cut into powder. After incubation in methanol for 24 h at 25°C, the mixture was centrifuged at 1.2 × 10^4^ r/min for 5 min. Transfer 800 μL of supernatant into another clean centrifuge tube and evaporate nitrogen at 40°C. The residue was then redissolved in 50 mL of mobile phase for LC-MS/MS analysis.

There was good performance, with limits of detection and quantification of 0.3 and 1.0 pg/mg for T, and 0.2 and 0.5 pg/mg for P, respectively. The recovery, intra-day, and inter-day coefficients of variation met the requirements. Analyses were conducted at the Jiangsu Provincial Key Laboratory of Biomaterials and Devices, Southeast University.

### 2.4 Statistical analysis

SPSS 25.0 software was used for data analysis. Normality of continuous variables was assessed via the Shapiro–Wilk test. Normally distributed data are presented as mean ± standard deviation, while non-normally distributed data are expressed as median (interquartile range, IQR). Group comparisons for normally distributed continuous variables utilized independent samples t-tests, whereas non-parametric Mann–Whitney U tests were applied to non-normally distributed data. Receiver operating characteristic (ROC) curve analysis was conducted to evaluate the predictive efficacy and the cutoff values of T and P for stroke risk. Categorical variables are reported as frequencies and percentages, with between-group comparisons performed using chi-square tests. A two-tailed *p* < 0.05 was considered statistically significant.

## 3 Results

### 3.1 Comparison of T and P expression levels between the two groups

The T and P levels of hair before the onset of stroke were significantly lower in the stroke group compared to the healthy control group (T: 3.60 vs. 6.90 pg/mg, *Z* = −3.52, *p* = 0.001; P: 5.85 vs. 9.80 pg/mg, *Z* = −3.08, *p* = 0.002). The median reductions in T and P levels were 48% and 40%, respectively (Table 2).

**Table 2:** Comparison of T, P levels between the two groups [M (IQR)].

Group	T (pg/mg)	P (pg/mg)
Stroke group (*n* = 48)	3.60 (3.35)	5.85 (5.65)
Control group (*n* = 35)	6.90 (4.70)	9.80 (10.40)
*Z*	−3.52	−3.08
*P*	0.001	0.002

### 3.2 Comparison of T and P expression levels in men between the two groups

Among male subjects, the T and P levels of hair before the onset of stroke were lower in the stroke group than in the control group. T levels were significantly reduced in the stroke group (3.90 vs. 6.80 pg/mg, *Z* = −2.39, *p* = 0.017), representing a 43% reduction. Although P levels were lower in the stroke group (6.90 vs. 8.70 pg/mg), this difference was not statistically significant (*p* = 0.195) (Table 3).

**Table 3:** Comparison of T, P levels in men between the two groups [M (IQR)].

Group	T (pg/mg)	P (pg/mg)
Stroke group (*n* = 48)	3.90 (3.15)	6.90 (10.90)
Control group (*n* = 35)	6.80 (3.70)	8.70 (6.40)
*Z*	−2.39	−1.30
*P*	0.017	0.195

### 3.3 Comparison of T and P expression levels in women between the two groups

Among female subjects, the T and P levels of hair before the onset of stroke were significantly lower in the stroke group than in the control group (T: 4.00 vs. 7.10 pg/mg, *Z* = −2.83, *p* = 0.005; P: 3.95 vs. 8.60 pg/mg, *Z* = −3.48, *p* < 0.001). The median reductions were 44% for T and 54% for P. The reduction in P was particularly pronounced and demonstrated higher statistical significance (*p* < 0.001) compared to T (*p* = 0.005) (Table 4).

**Table 4:** Comparison of T, P levels in women between the two groups [M (IQR)].

Group	T (pg/mg)	P (pg/mg)
Stroke group (*n* = 48)	3.15 (4.30)	5.70 (2.93)
Control group (*n* = 35)	8.90 (19.25)	18.55 (25.63)
*Z*	−2.83	−3.48
*P*	0.005	0.001

### 3.4 ROC analysis of HPG axis hormones in stroke onset prediction

**Figure 1:**
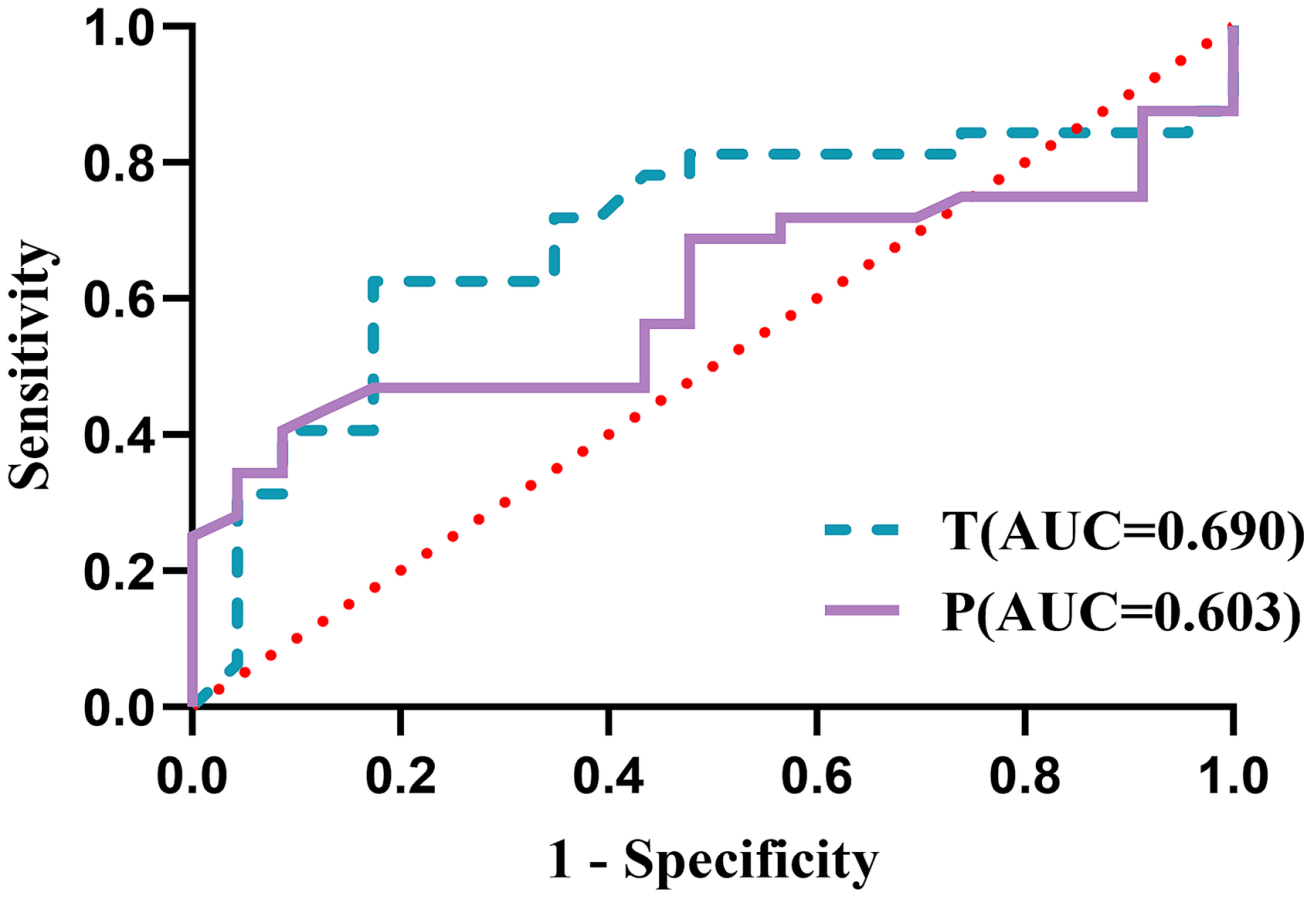
ROC curves for HPG axis hormones in men.

**Figure 2:**
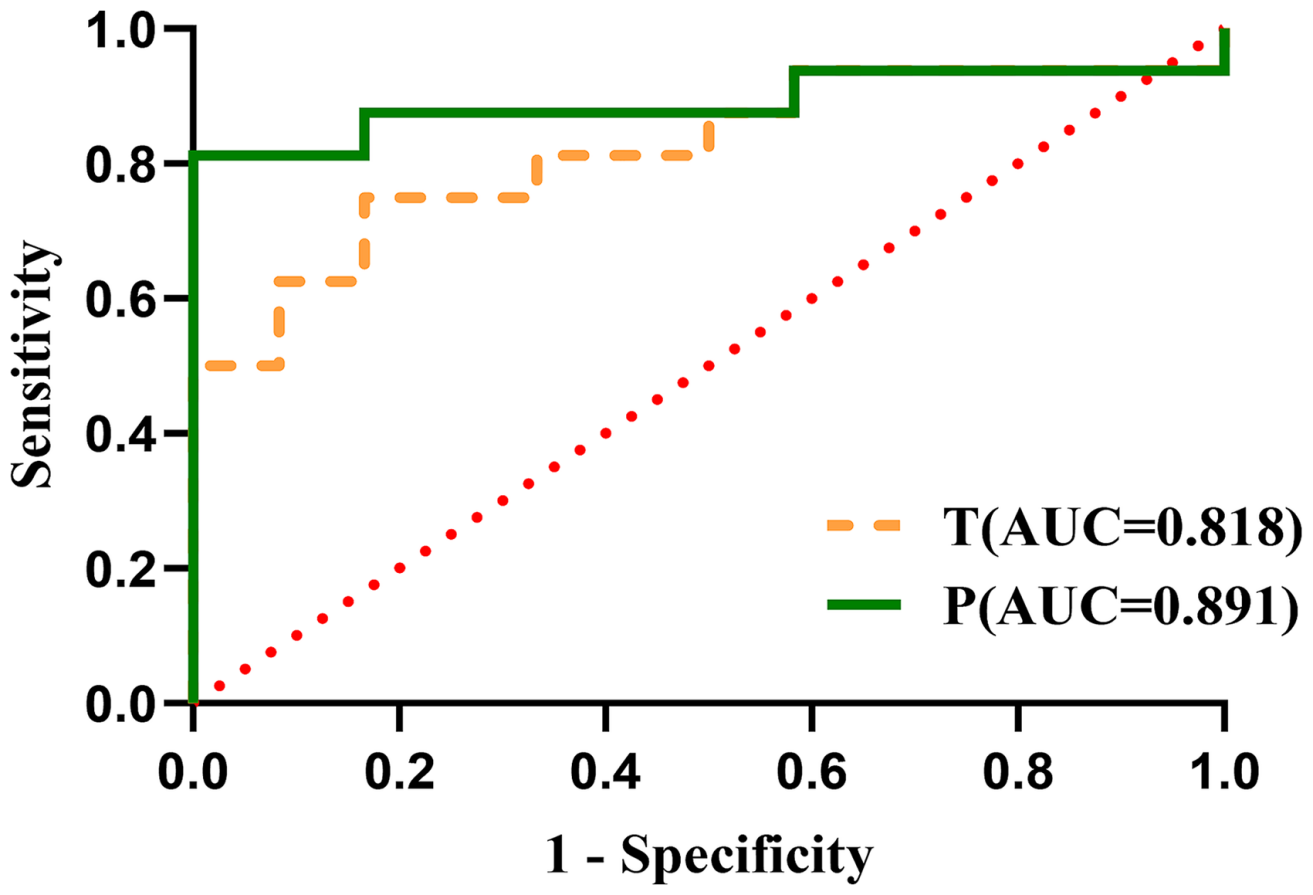
ROC curves for HPG axis hormones in women.

The results of ROC curve showed that the cut-off value of T level in men’s hair before the onset of stroke was 4.35 pg/mg, the area under the curve (AUC) was 0.690 (0.545, 0.835), the sensitivity was 62.50%, and the specificity was 82.61%. The cut-off value of T level in women’s hair before the onset of stroke was 5.00 pg/mg, the AUC was 0.818 (0.658, 0.978), the sensitivity was 75.00%, and the specificity was 83.33%. The cut-off value of P level in women’s hair before the onset of stroke was 8.00 pg/mg, the AUC was 0.891 (0.754, 1.000), the sensitivity was 81.25%, and the specificity was 100.00% (Table 5; Figures 1, 2).

**Table 5:** The cutoff value, sensitivity, and specificity of HPG axis hormones.

Hormone	AUC (95%CI)	*p* value	The cut-off value	Sensitivity	Specificity
T (men)	0.690 (0.545, 0.835)	0.017	<4.35	62.50%	82.61%
P (men)	0.603 (0.453, 0.753)	0.195	<4.85	40.63%	91.30%
T (women)	0.818 (0.658, 0.978)	0.005	<5.00	75.00%	83.33%
P (women)	0.891 (0.754, 1.000)	0.000	<8.00	81.25%	100.00%

## 4 Discussion

Existing studies (5, 16) indicate that the mean age of onset for ischemic stroke is 57 years in males and 59 years in females. Given the sharp decline in P levels among women after age 40 and significant fluctuations in T levels in men beyond 80 years, this study restricted enrollment to participants aged 50–80 years to reduce confounding effects.

Our findings demonstrate significantly lower pre-stroke hair T and P levels in the stroke cohort compared to healthy controls (*p* < 0.05), suggesting potential predictive utility of HPG axis hormones for stroke risk. This aligns with prior research: Kloner et al. (17) found in their review that low T levels increase the risk of cardiovascular and cerebrovascular diseases, while Molenberg et al. (18) employed Mendelian randomization to establish associations between HPG axis dysregulation and aneurysmal subarachnoid hemorrhage. Therefore, detecting the expression levels of HPG axis hormones may have a certain indicative effect on the risk of stroke onset.

Considering that the HPG axis hormone level is greatly affected by gender (16), T and P levels were analyzed separately by gender. The results showed that male stroke patients had significantly lower pre-stroke hair T levels compared to healthy controls (*p* < 0.05), whereas female stroke patients exhibited significantly lower pre-stroke hair T and P levels (*p* < 0.05). This suggests that T levels may predict stroke risk in males, while both T and P levels are predictive in females.

In males, T levels decline with age (17). Ho et al. (19) demonstrated that low T levels correlate with reduced survival rates, and males with low T levels at admission exhibiting higher mortality rates. Which potentially mediated by T’s neuroprotective roles in mitigating oxidative stress, enhancing cerebral antioxidant capacity, and inhibiting neuronal apoptosis (4). Yeap et al. (20) identified T as an independent predictor of stroke in males, with low dihydrotestosterone (DHT) also serving as an independent risk factor. However, serum-based hormone measurements in these studies are prone to daily-life confounders, limiting their clinical utility for establishing reliable cutoff values. Our research findings are consistent with their findings, and through ROC curve evaluation, we identified a T cutoff value of 4.35 pg/mg in male hair samples for stroke risk prediction, with an AUC of 0.690 (95% CI 0.545–0.835), sensitivity of 62.50%, and specificity of 82.61%. While testosterone replacement therapy (TRT) may improve low T levels in older patients, Loo et al. (21) identified that TRT in elderly males with low T levels may increase cardiovascular and cerebrovascular diseases, necessitating further investigation into endogenous versus exogenous hormone effects.

Epidemiological studies consistently demonstrate significant gender disparities in ischemic stroke incidence, with premenopausal females exhibiting markedly lower rates compared to age-matched males (22). This protective effect substantially diminishes following menopause, as evidenced by a pronounced increase in stroke incidence among postmenopausal females (22). Converging evidence from preclinical and clinical investigations indicates that exogenous progesterone administration confers significant neuroprotection (23–25). In experimental models, progesterone effectively reduces cerebral infarct volume and enhances functional recovery post-stroke (26). The neuroprotective mechanisms of progesterone appear multifaceted, potentially mediated through several key pathways: attenuation of inflammatory responses (27), preservation of blood–brain barrier (BBB) integrity (28), amelioration of mitochondrial dysfunction (29), mitigation of oxidative damage, and facilitation of myelin repair (30, 31). Collectively, these findings underscore progesterone’s substantial therapeutic potential as a cytoprotective agent for mitigating secondary brain injury and improving neurological outcomes after ischemic insult (24). However, no prior studies have examined pre-stroke P levels in relation to stroke risk. Our study identified significantly lower pre-stroke hair P levels in female stroke patients versus controls (*p* < 0.01), and identified a P cutoff value of 8.00 pg/mg in female hair samples for stroke risk prediction, with an AUC of 0.891 (95% CI 0.754–1.000), sensitivity of 81.25%, and specificity of 100.00%. At the same time, some studies (32) have shown that reduced E_2_ and P levels may not solely account for elevated stroke risk in women. The adverse effect of T on cardiovascular and cerebrovascular diseases, resulting in elevated testosterone-to-estradiol (T/E2) ratios may contribute to postmenopausal stroke incidence, which requires further research. Quantification of pre-stroke hair testosterone levels in female patients revealed significant disparities versus healthy controls (*p* < 0.01). And ROC curve analysis identified an optimal T cutoff of 5.00 pg/mg (AUC 0.818, 95% CI 0.658–0.978; sensitivity 75.00%, specificity 83.33%) for stroke risk stratification.

The analysis of hair HPG axis hormone levels offers unique advantages, including its cumulative nature, long-term retrospective assessment capacity, and non-invasiveness. Standardized detection methods based on LC–MS/MS can serve as a reference for clinical screening of high-risk stroke populations. This approach facilitates the detection of cryptogenic stroke and consequently contributes to improved patient outcomes. With future cost optimization and standardized implementation, hair hormone analysis holds promise as a valuable tool for primary stroke prevention.

### 4.1 Limitations

However, there are several limitations to this study. Due to technical limitations, the investigation focused solely on T and P; future studies ought to incorporate estradiol (E2), follicle-stimulating hormone (FSH), and DHT to further elucidate the impact of the HPG axis on stroke. As a pilot investigation, it was not feasible to comprehensively adjust for all confounding variables—including hypertension, dietary patterns, and physical activity levels—which will be rigorously accounted for in subsequent studies. The derived cut-off values for HPG-axis hormones in predicting stroke risk demonstrate potential clinical utility; however, their external validity requires rigorous verification through multicenter randomized controlled trials with larger cohorts.

## 5 Conclusion

In conclusion, this preliminary report is the first to suggest that HPG axis hormones such as T and P in hair could have predictive value in screening for stroke risk.

## Data Availability

The original contributions presented in the study are included in the article/Supplementary material, further inquiries can be directed to the corresponding authors.
